# Preoperative thrombocytopenia as a predictor of cancer-associated thromboembolism in gastric cancer patients undergoing gastrectomy

**DOI:** 10.3389/fonc.2025.1585201

**Published:** 2025-07-16

**Authors:** Moonki Jung, Song Ee Park, Jin Hwa Choi, Jeong Eun Kim, In Gyu Hwang

**Affiliations:** ^1^ Department of Internal Medicine, Chung-Ang University Gwang-myeong Hospital, Gwang-myeong, Republic of Korea; ^2^ Division of Medical Oncology, Department of Internal Medicine, Seoul St. Mary’s Hospital, College of Medicine, The Catholic University of Korea, Seoul, Republic of Korea; ^3^ Chung-Ang University Integrated Oncology and Palliative Care Research Institute, Seoul, Republic of Korea; ^4^ Department of Radiation Oncology, Chung-Ang University College of Medicine, Seoul, Republic of Korea; ^5^ Department of Oncology, Asan Medical Center, University of Ulsan College of Medicine, Seoul, Republic of Korea; ^6^ Department of Internal Medicine, Chung-Ang University College of Medicine, Seoul, Republic of Korea

**Keywords:** thrombocytopenia, thromboembolism, gastric cancer, gastrectomy, prognosis

## Abstract

**Purpose:**

Cancer-associated thromboembolism (CAT) is a major complication in gastric cancer, impacting patient outcomes. This study aimed to evaluate preoperative thrombocytopenia as a risk factor for CAT in gastric cancer patients undergoing gastrectomy.

**Materials and methods:**

A retrospective analysis was conducted on 610 gastric cancer patients who underwent D2 gastrectomy between 2005 and 2017. The incidence of CAT and its association with preoperative thrombocytopenia, cancer stage, and recurrence were analyzed. Prognostic factors for CAT and survival were assessed using Kaplan-Meier analysis and Cox regression models.

**Results:**

The median follow-up was 67.0 months. Among the study participants, 5.7% (n=35) developed CAT. Preoperative thrombocytopenia was present in 71 patients (11.6%) and older age (≥65 years) was noted in 308 patients (50.1%). The 5-year incidence rate of CAT was 16.3% in patients with preoperative thrombocytopenia, compared to 4.8% in those with normal platelet counts. Patients with preoperative thrombocytopenia had a higher incidence of CAT compared to those with normal platelet counts (HR = 3.180, 95% CI 1.527-6.623, p = 0.002). Multivariate analysis revealed that thrombocytopenia (HR = 2.202, 95% CI 1.029-4.711, p = 0.042), older age (HR 2.484, 95% CI 1.166-5.290, p = 0.018), stage IV (HR = 2.966, 95% CI 1.106-8.466, p = 0.038) were independent poor prognostic factors for preoperative CAT in gastric cancer patients. Additionally, 29.7% of patients experienced cancer relapse, and 26.1% died during post-treatment follow-up. the patients who developed thromboembolisms had a significantly shorter 5-year OS rate compared with those who did not (56.6% vs. 76.1%, HR = 2.277, 95% CI = 1.376–3.767, p = 0.001).

**Conclusions:**

Preoperative thrombocytopenia is a significant predictor of CAT in gastric cancer patients. Along with advanced stage and older age, it increases thromboembolic risk and worsens survival. Further studies are needed to refine predictive models and optimize management strategies.

## Introduction

Gastric cancer is a prevalent malignancy characterized by high morbidity and the fourth-highest mortality rate worldwide (1). Among the complications associated with gastric cancer, the thromboembolism is a significant concern due to its potential impact on patient outcomes and survival.

The incidence of cancer-associated venous thromboembolism, including pulmonary embolism (PE) and deep vein thrombosis (DVT), has been closely associated with survival outcomes in cancer patients (2, 3). Cancer-associated thromboembolism has been linked to various risk factors. Among these factors, the primary site of the tumor has emerged as a significant determinant of cancer-associated thromboembolism. Gastric cancer, in particular, has been identified as one of the most thrombogenic tumor types (4).

Many studies have demonstrated that cancer-associated thromboembolism is an independent prognostic factor for poor survival in patients with cancer. This correlation has been observed in gastric cancer (5) and other gastrointestinal malignancies, such as colon (6, 7), pancreatic (8), and lung cancer (9). Therefore, it is crucial to identify the risk factors associated with thromboembolism related to gastric cancer.

Cancer patients with thrombocytopenia are risk for developing thrombosis, a complication that poses significant clinical challenges (10). While low platelet counts typically increase the risk of bleeding, in the context of malignancy, various factors such as pro-coagulant activity of tumor cells, chemotherapy, and inflammation can lead to thrombotic events despite thrombocytopenia. This thrombosis, often associated with poor prognosis, necessitates careful clinical evaluation and management. Early recognition and appropriate treatment are essential to mitigate the risk of life-threatening complications in these patients, even before definitive diagnostic confirmation.

Therefore, this study investigated the occurrence of cancer-associated thromboembolism in a cohort of patients with gastric cancer who underwent gastrectomy as part of their treatment. The study aimed to identify the prognostic factors associated with the incidence of thromboembolism in patients with gastric cancer post-gastrectomy.

## Materials and methods

### Patient cohort

A total of 610 patients with primary gastric cancer who had undergone D2 radical gastrectomy at the Chung-Ang University Hospital (Seoul, Korea) between January 2005 and December 2017 were enrolled in this study. The Chung-Ang University Hospital’s pathologist histologically examined all the cases. Cancer variables, including stage and grade, were retrospectively collected. The diagnosis of gastric cancer was confirmed by pathological staining. The cancer staging was performed according to the American Joint Committee on Cancer, 7th edition (11). This study was approved by the Institutional Review Board of Chung-Ang University Hospital (IRB number: 2212-017-19450).

### Study outcomes

The study’s primary outcome is to indentify risk factors for cancer-associated thromboembolism; the secondary outcome is to idenfify risk factors for patients’ disease free survival(DFS) and overall survival (OS).

The primary outcome measure was the incidence of cancer-associated thromboembolism, including symptomatic or incidentally found PE, DVT of the upper or lower limbs, and visceral venous thrombosis of patients who had undergone gastronomy for gastric cancer. The thromboembolism was confirmed by computed tomography. The patients’ time of death, cancer progression time, or time to develop cancer-associated thromboembolism post-gastrectomy were determined for all cases. In our study, thrombocytopenia was defined as a platelet count of less than 150,000/mL.

### Statistical analysis

All statistical analyses were performed using the Statistical Package for Social Sciences (SPSS) version 26.0 (IBM Statistical Software™, IBM Corp., Armonk, NY, USA), including the patients’ baseline characteristics and prognostic factors. The correlation between prognostic factors involved thrombocytopenia, stage and older age and the CAT was examined using Cox proportional hazards regression modeling The correlation between death and the CAT was examined using Cox proportional hazards regression modeling and the Kaplan–Meier survival curve. A *p* value < 0.05 was considered statistically significant. GraphPad Prism 9.0 (GraphPad Inc., La Jolla, CA, USA) was used to generate the survival curves.

## Results

### Patients’ baseline characteristics

A total of 610 patients were included in this study, among whom 35 (5.7%) developed cancer-associated thromboembolism (CAT). The median age of the cohort was 65 years (range: 28–90), with a significantly higher median age observed in the CAT-positive group (68 years) compared to the CAT-negative group (64 years, P = 0.009). Patients aged ≥65 years were more likely to develop CAT (71.4% vs. 49.2%, P = 0.011). No significant difference was observed in sex distribution between the two groups (P = 0.598) (**Table 1**).

**Table 1:** Patients’ baseline characteristics.

Characteristics	Total (n=610)	CAT (–) (n=575)	CAT (+) (n=35)	P value
Demographic Value				
Age - years				
median	65	64	68	0.009
range	28-90	28-90	50-86	
Age ≥65 years	308 (50.5%)	283 (49.2%)	25 (71.4%)	0.011
Sex				
Male	411 (67.4%)	386 (67.1%)	25 (71.4%)	0.598
Female	199 (32.6%)	189 (32.9%)	10 (28.6%)	
Height (cm), mean ± SD	161.9 ± 9.0	162.0 ± 9.0	1604 ± 10.2	0.445
Weight (kg), mean ± SD	62.2 ± 11.9	62.3 ± 11.9	60.6 ± 3.1	0.810
BMI, kg/m^2^, mean ± SD	23.6 ± 3.5	23.6 ± 3.5	23.4 ± 3.1	0.281
Hematologic Value				
Platelet < 150,000/mL	71 (11.6%)	61 (10.6%)	10 (28.6%)	0.001
Cancer specific variables				
Gastric cancer stage				
Stage I	360 (59.0%)	340 (59.1%)	22 (62.9%)	0.003
Stage II	98 (16.1%)	95 (16.5%)	3 (8.6%)	
Stage III	121 (19.8%)	117 (20.3%)	4 (11.4%)	
Stage IV	29 (4.8%)	23 (4.0%)	6 (17.1%)	
Adjuvant chemotherapy				
No	370 (60.7%)	348 (60.5%)	22 (62.9%)	0.784
Yes	240 (39.3%)	227 (39.5%)	13 (37.1%)	
Khorana Score variables				
BMI ≥ 35 kg/m^2^	1 (0.2%)	1 (0.2%)	0 (0%)	0.805
Hemoglobine < 10 g/dL	84 (13.8%)	80 (13.9%)	4 (11.4%)	0.679
WBC ≥ 11000/mL	258 (42.3%)	244 (42.4%)	14 (40.0%)	0.777
Platelet ≥ 350,000/mL	48 (4.9%)	47 (8.2%)	1 (2.9%)	0.257
Khorana score Intermediate (≤2)	269 (44.1%)	252 (43.8%)	17 (48.6%)	0.583
Khorana score High (≥3)	341 (55.9%)	323 (56.2%)	18 (51.4%)	

CAT. Cancer associated thromboembolism, BMI, body mass index; SD, standard deviation; WBC, white blood cell count.

Regarding hematologic parameters, thrombocytopenia (platelet <150,000/mL) was more prevalent in the CAT-positive group (28.6% vs. 10.6%, P = 0.001). Cancer-specific variables showed a significant association with CAT, particularly in patients with advanced-stage gastric cancer. The incidence of CAT was highest in stage IV patients (20.7%), followed by stage I (6.1%), stage III (3.3%), and stage II (3.1%) (P = 0.002). Adjuvant chemotherapy was not significantly associated with CAT occurrence (P = 0.784).

For Khorana score components, no significant differences were observed between the CAT-positive and CAT-negative groups in terms of BMI ≥35 kg/m² (P = 0.805), hemoglobin <10 g/dL (P = 0.679), WBC ≥11,000/mL (P = 0.777), and platelet ≥350,000/mL (P = 0.257).

### Incidence of cancer-associated thromboembolism

The incidence of cancer-associated thromboembolism in the 610-patient cohort post- gastrectomy was 5.7% (35 patients). Among these cases, two patients presented with extremity DVT, while 19 patients were diagnosed with splanchnic vein thrombosis (SVT). Additionally, 10 patients experienced PE, and four exhibited other embolic events, including three cases of brain infarction and one of myocardial infarction.

**Figure 1:**
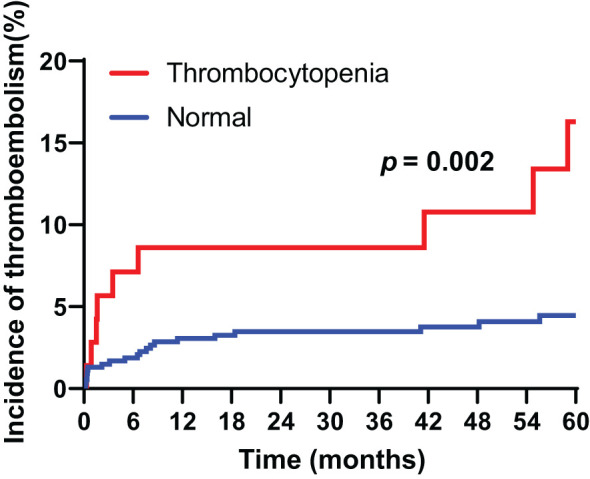
Cumulative incidence of cancer-associated thromboembolism in patients with gastric cancer according to thrombocytopenia.

The cumulative incidence of venous thromboembolism during the follow-up period (median 67.0 months) reached 5.7%. After gastrectomy. The 5-year incidence rate of cancer associated thrombosis was 16.3% in patients with preoperative thrombocytopenia, compared to 4.8% in those with normal platelet counts. Patients with preoperative thrombocytopenia had a higher incidence of cancer-associated thrombosis compared to those with normal platelet counts (HR = 3.180, 95% CI 1.527-6.623, p = 0.002) (**Figure 1**).

Furthermore, the cumulative incidence of venous thromboembolism was 1.8%, 2.3%, and 2.8% at 12, 24, and 36 months, respectively. The cumulative incidence of thromboembolism according to cancer stage was 6.1%, 3.1%, 3.3%, and 20.7% in stages I, II, III, and IV, respectively; these differences were statistically significant (*p* = 0.002). The results indicated no statistically significant differences in cumulative incidences of thromboembolism between cancer stage groups as follows: stage I, *p* = 0.723; stage II, *p* = 0.973; stage III, *p* = 0.795; and stage IV, *p* = 0.063.

Multivariate analysis revealed that both thrombocytopenia (HR = 2.202, 95% CI 1.029-4.711, p = 0.042), older age (HR 2.484, 95% CI 1.166-5.290, p = 0.018) and stage IV (HR = 2.996, 95% CI 1.106-8.466, p = 0.038) were independent poor prognostic factors for preoperative CAT in gastric cancer patients (**Table 2**).

**Table 2:** Univariate and multivariate analysis of the incidence of cancer-associated thromboembolism (CAT) by risk factor.

Risk factor	Univariate analysis			Multivariate analysis		
	HR	95% CI	P value	HR	95% CI	P value
Demographic Value						
Age ≥ 65	2.580	1.217-5.468	0.013	2.484	1.166-5.290	0.018
Male	0.817	0.384-1.736	0.599			
Hematologic variable						
Platelet < 150,000/mL	3.370	1.545-7.352	0.002	2.202	1.029-4.711	0.042
Cancer specific variables						
Gastric cancer stage			0.003			0.011
Stage I	Reference					
Stage II	0.501	0.150-1.673	0.261	0.422	0.124-1.433	0.167
Stage III	0.685	0.235-1.995	0.448	0.425	0.130-1.342	0.145
Stage IV	6.109	2.408-15.498	<0.001	2.996	1.106-8.466	0.038
Adjuvant chemotherapy	0.943	0.475-1.872	0.866			
Khorana Score variables						
BMI ≥ 35 kg/m^2^	0.050	0.000-5.567E+16	0.887			
Hemoglobin < 10 g/dL	0.798	0.274-2.322	0.679			
WBC ≥ 11000/mL	0.883	0.449-1.736	0.718			
Platelet ≥ 350,000/mL	0.368	0.050-2.688	0.324			

BMI, body mass index; CI, confidence interval; HR, hazard ratio; WBC, white blood cell count.

### Survival

**Figure 2:**
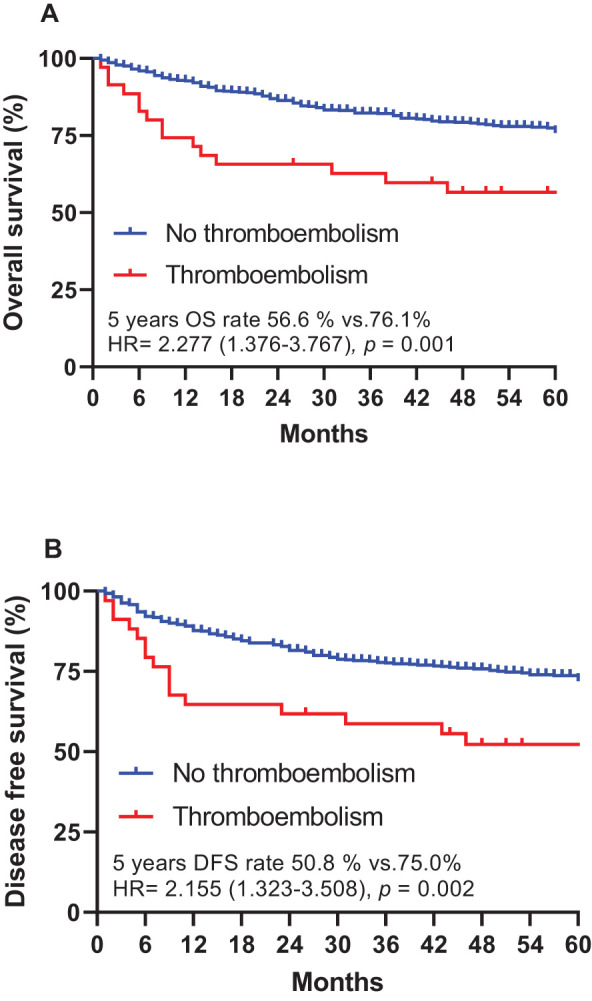
Overall survival **(A)** and disease-free survival **(B)**, stratified by cancer-associated thromboembolism (CAT) in patients with gastric cancer. DFS, disease-free survival; HR, hazard ratio; OS, overall survival.

The study’s cutoff for analyses was January 2005, and the median follow-up duration was 67.0 months (range 0.1–155.0 months). The 5-year OS rate was 75.0%, and the 5-year disease-free survival (DFS) rate was 70.9%. Of the 610 patients, 181 (29.7%) had cancer relapse after treatment, and 159 (26.1%) died. The patients who developed thromboembolism had a significantly shorter 5-year DFS rate compared with those who did not (50.8% vs. 72.0%, HR = 2.155, 95% CI = 1.323–3.508, *p* = 0.002). In addition, the patients who developed thromboembolisms had a significantly shorter 5-year OS rate compared with those who did not (56.6% vs. 76.1%, HR = 2.277, 95% CI = 1.376–3.767, *p* = 0.001) (**Figure 2**).

In univariate analysis, age ≥65 years was significantly associated with poor overall survival (HR: 2.251, 95% CI: 1.618–3.133, P < 0.001), which remained significant in multivariate analysis (HR: 2.094, 95% CI: 1.494–2.934, P < 0.001). Advanced gastric cancer stage was a strong predictor of worse survival outcomes, with stage IV patients having the highest mortality risk (HR: 21.839, 95% CI: 13.007–36.850, P < 0.001 in univariate analysis; HR: 20.278, 95% CI: 11.433–35.966, P < 0.001 in multivariate analysis).

Cancer-associated thromboembolism was associated with increased mortality risk in univariate analysis (HR: 2.277, 95% CI: 1.376–3.767, P = 0.001), but this association did not reach statistical significance in multivariate analysis (HR: 1.684, 95% CI: 0.956–2.966, P = 0.071). Other factors, such as hemoglobin <10 g/dL and WBC ≥11,000/mL, showed statistical significance in univariate analysis but were not significant predictors in multivariate analysis (**Table 3**).

**Table 3:** Univariate and multivariate analysis of the overall survival.

Risk factor	Univariate analysis			Multivariate analysis		
	HR	95% CI	P value	HR	95% CI	P value
Demographic Value						
Age ≥ 65	2.251	1.618-3.133	<0.001	2.094	1.494-2.934	<0.001
Male	0.935	0.673-1.301	0.691			
Hematologic variables						
Platelet < 150,000/mL	1.250	0.797-1.960	0.332			
Cancer specific variables						
Gastric cancer stage			<0.001			<0.001
Stage I	Reference			Reference		
Stage II	1.923	1.108-3.335	0.020	1.928	1.059-3.513	0.032
Stage III	9.492	6.425-14.024	<0.001	9.843	6.136-15.789	<0.001
Stage IV	21.839	13.007-36.850	<0.001	20.278	11.433-35.966	<0.001
Cancer thromboembolism	2.277	1.376-3.767	0.001	1.684	0.956-2.966	0.071
Khorana Score variables						
BMI ≥ 35 kg/m^2^	0.050	0-16993705	0.765			
Hemoglobin < 10 g/dL	2.223	1.531-3.227	<0.001	0.976	0.656-1.452	0.903
WBC ≥ 11000/mL	0.397	0.272-0.564	<0.001	0.992	0.645-1.526	0.971
Platelet ≥ 350,000/mL	1.550	0.937-2.564	0.088	0.781	0.456-1.338	0.368

BMI, body mass index; CI, confidence interval; HR, hazard ratio; WBC, white blood cell count.

## Discussion

This study investigated the incidence and risk factors of cancer-associated thromboembolism (CAT) in a cohort of gastric cancer patients who underwent gastrectomy. The overall incidence of CAT was 5.7%, with a significantly higher occurrence in patients with preoperative thrombocytopenia. This finding reinforces the role of thrombocytopenia as a potential predictor of thromboembolic complications in gastric cancer patients.

Thrombocytopenia is a common condition in cancer patients due to multiple factors, including disease burden and chemotherapy-induced toxicity (12). It has frequently been reported in conjunction with CAT events, and our study further supports its association with increased thromboembolic risk. The 5-year incidence rate of CAT in patients with preoperative thrombocytopenia was markedly higher at 16.3% compared to 4.8% in those with normal platelet counts. Multivariate analysis confirmed thrombocytopenia as an independent risk factor for CAT, along with older age and stage IV disease. These findings align with prior studies suggesting that thrombocytopenia may paradoxically contribute to a hypercoagulable state, possibly due to increased circulating procoagulant microparticles from platelet destruction, enhanced thrombopoiesis, or tumor-driven inflammation (13).

Although thrombocytopenia is typically associated with bleeding risk, several biological mechanisms may explain its contribution to thrombotic susceptibility in cancer. Platelet fragmentation can release microparticles that serve as catalytic surfaces for thrombin generation (14). In response to low platelet counts, the bone marrow may generate immature, hyperreactive platelets. Additionally, tumor-secreted cytokines can activate coagulation pathways independently of platelet numbers (15).

This study also examined the impact of gastric cancer stage on CAT incidence. A statistically significant difference was observed across cancer stages, with stage IV patients exhibiting the highest thromboembolic incidence at 20.7%. This underscores the importance of considering cancer stage when assessing thromboembolic risk and highlights the necessity for proactive risk management strategies, particularly in patients with advanced disease.

Regarding the impact of CAT on patient survival, our results revealed a significantly shorter 5-year overall survival (OS) rate in patients with thromboembolism compared to those without. This highlights the detrimental impact of CAT on long-term prognosis and underscores the need for early identification and intervention to improve patient outcomes.

Notably, a portion of patients within our study population developed arterial thrombotic complications, including strokes and heart attacks, even in the context of low platelet counts. This observation suggests that in gastric cancer, thrombocytopenia may reflect underlying platelet hyperactivity or a prothrombotic state, rather than simply indicating a bleeding predisposition. In these individuals, the use of antiplatelet therapy alongside low molecular weight heparin (LMWH) may hold therapeutic promise (16). Nonetheless, due to the elevated hemorrhagic risk associated with gastric tumors—particularly those with mucosal involvement—such dual-agent regimens should be carefully tailored to each patient (17). Further prospective trials are essential to clarify the safety and potential benefits of these combined interventions in this challenging clinical scenario.

Despite its valuable insights, this study has limitations. As a single-center study, potential selection bias cannot be excluded, and the findings may not be fully generalizable to broader populations. We acknowledge that external validation is necessary to strengthen the robustness of our findings. Multicenter, prospective studies are warranted to validate our predictive model and ensure its applicability across diverse clinical settings.

Furthermore, while we identify thrombocytopenia as a significant risk factor, the study was not designed to investigate underlying biological mechanisms. Future translational studies are needed to elucidate the molecular and cellular pathways that link thrombocytopenia to thrombosis in gastric cancer.

In conclusion, this study identifies key predictive factors for CAT in gastric cancer patients post-gastrectomy, including preoperative thrombocytopenia, advanced cancer stage and older age. These findings support the need for personalized risk stratification and targeted preventive strategies to reduce the burden of CAT in this population. Furthermore, careful consideration of antithrombotic strategies—balancing thrombotic and bleeding risks—is essential, particularly in patients with arterial events and thrombocytopenia. Further research is essential to optimize thromboprophylactic approaches and improve clinical outcomes in gastric cancer patients at risk for thromboembolism.

## Data Availability

The original contributions presented in the study are included in the article/supplementary material. Further inquiries can be directed to the corresponding author.
